# Effects of Dietary Barley Starch Contents on the Performance, Nutrient Digestion, Rumen Fermentation, and Bacterial Community of Fattening *Hu* Sheep

**DOI:** 10.3389/fnut.2021.797801

**Published:** 2022-01-27

**Authors:** Xiaowen Ma, Wenjing Zhou, Tongqing Guo, Fei Li, Fadi Li, Tao Ran, Zhian Zhang, Long Guo

**Affiliations:** State Key Laboratory of Grassland Agro-ecosystems, Key Laboratory of Grassland Livestock Industry Innovation, Ministry of Agriculture and Rural Affairs, Engineering Research Center of Grassland Industry, Ministry of Education, College of Pastoral Agriculture Science and Technology, Lanzhou University, Lanzhou, China

**Keywords:** corn starch, barley starch, nutrient digestibility, rumen health, sheep

## Abstract

The objective of this experiment was to investigate the effects of substituting corn starch (CS) with barley starch (BS) on the growth performance, nutrient digestion, rumen fermentation, and bacterial community of fattening *Hu* sheep. Seventy-two *Hu* lambs with similar initial body weight (BW, 29.70 ± 1.70 kg) were randomly assigned to four treatments, with 18 lambs per group. The four experimental diets have identical starch contents but with different starch sources as 100% starch from corn (BS-0), 33% starch from barley and 67% starch from corn (BS-33), 67% starch form barley and 33% starch from corn (BS-67), and 100% starch from barley (BS-100). All lambs were reared in individual units and fed high-concentrate diets (85% concentrate in diets based on dry matter [DM]). The experimental period included 7 days for adaptation and 63 days for data collection. Sixteen ruminal cannulated *Hu* sheep were divided into 4 groups and received the four experimental diets to determine the dynamics of ruminal pH. The average daily gain (ADG), and BW gain of lambs linearly decreased (*p* < 0.05), whereas the feed to gain ratio linearly increased (*p* < 0.05) with increasing dietary proportions of BS. Digestibility of DM, organic matter, neutral detergent fiber, acid detergent fiber, starch, and gross energy (GE) decreased (*p* < 0.05) with increasing dietary BS contents. Ruminal mean pH decreased (*p* < 0.05) with increasing proportions of dietary BS, accompanied with linearly increased (*p* < 0.05) time and area of ruminal pH below 5.80 or 5.60. Increasing dietary proportions of BS linearly decreased (*p* < 0.05) the molar proportion of acetate, but linearly increased (*p* < 0.05) the molar proportion of propionate. Sheep of the BS-0 and BS-33 treatments had a less (*p* < 0.05) relative abundance of *Selenomonas ruminantium* than that of sheep of the BS-67 treatment, but a greater (*p* < 0.05) relative abundance of *Ruminococcus albus* than that of sheep of the BS-100 treatment (*p* < 0.05). In conclusion, feeding a high-concentrate corn-based diet for fattening *Hu* sheep improved the performance and rumen fermentation parameters when compared to the barley-based diet.

## Introduction

Using cereal grain to increase the dietary starch contents is a commonly used strategy to improve the performance and feed efficiency of sheep (1), dairy cow (2), and beef cattle (3) under the modern intensive ruminant production systems. Attention should be paid while choosing cereal grain during diet formulation, as the ruminal degradation rates of starch from different cereal grains can be quite variable. For instance, the degradation rate of starch in corn was lower than that in barley after dry-rolled (4, 5). The lower degradation rate of corn starch (CS) compare to the other sources of starch in diets could decrease the risk of subacute ruminal acidosis (SARA) in high production ruminates (6, 7). Corn grain is an important energy source and is widely used in the ruminant livestock industry in China. However, the price of corn grain raised nearly 50% in China from 2020 to 2021, resulting in a dramatic increase in feed costs for ruminant production. Therefore, it is important to study alternative cereals such as barley or wheat to replace corn, so that to decrease the feed costs without affecting the performance of ruminants.

The barley grain that has a high content of crude protein (CP) but lower metabolize energy (90% of corn grain) than corn is commonly used for the substitution of corn. Surber et al. (8) found that replacing corn by barley increased the microbial nitrogen synthesis rate. However, the ruminal degradation rate of barley starch (BS) was greater than that of CS, which potentially increased the risk of SARA for dairy cows and beef cattle. This was supported by Emmanuel et al. (9) that the ruminal pH of lactating dairy cows decreased from 6.8 to 6.3 when the dietary barley proportions increased from 0 to 45%. Yahaghi et al. (10) also reported that the ruminal pH of lamb decreased from 6.39 to 5.8 when the substitution rate of dietary barley for corn increased from 25 to 84%.

The combination of grain sources differing in the rates of ruminal fermentation may affect the growth performance of ruminants. Haddad and Nasr (11) found that 20% barley for the lambs had a higher final body weight (BW) (34.3 vs. 32.2 kg) and average daily gain (ADG, 186 vs. 164 g/day) compared with the 10% group. Yahaghi et al. (12) reported that substitution of corn with 30% barley improved ruminal pH (6.05 vs. 5.95) compared with the substitution of corn with 70% barley for lambs. Johnson et al. (5) reported higher digestibilities of dry matter (DM), organic matter (OM), CP, neutral detergent fiber (NDF), and starch for the barley grain treatment (85.94% of diet) compared to the corn grain treatment (84.96% of diet), or the combination of barley and corn group (42.72% corn and 42.72% barley of diet). These studies revealed that the varied proportions of the barley and corn may change the content of rumen degradable starch and finally affect the rumen fermentation, nutrient digestibility, and performance of ruminant.

In the present study, we hypothesized that the substitution of CS with BS could decrease the ruminal pH and change the bacterial community of rumen, which might affect the nutrients digestibility and performance of fattening *Hu* sheep. In addition, we also intended to find out a feasible combination of corn and barley for fattening sheep production based on the performance and economic consideration.

## Materials and Methods

All procedures were approved by the guidelines formulated by the Biological Studies Animal Care and Use Committee of Gansu Province, China (2005–12).

### Animals, Diets, and Management

Seventy-two male *Hu* lambs of 3 months of age with similar initial BW (29.70 ± 1.70 kg) were randomly assigned into four treatments (18 lambs per treatment). The four experimental diets had identical starch content (25.5% of diet, DM basis) but varied in the percentage of CS replaced by BS, and were: 100% starch from corn (BS-0), 33% starch from barley and 67% starch from corn (BS-33), 67% starch from barley and 33% starch from corn (BS-67), and 100% starch from barley (BS-100). The experimental diets were offered as pelleted total mixed ration, the cereal grains of which were grounded through 4 mm sieve before pelleting the diet, and the formulation and chemical composition of the experimental diets were shown in Table 1. All lambs were raised in individual pens (0.65 × 1.50 × 1.10 m), fed *ad libitum*, and had free access to water. The experiment lasted 70 days, with 7 days for diet adaptation and 63 days for data collection.

**Table 1:** Ingredients and nutrient levels of experimental diets.

**Items**	**Treatments^a^**			
	**BS-0**	**BS-33**	**BS-67**	**BS-100**
Ingredients (% air-dry basis)				
Barley straw	15.00	15.00	15.00	15.00
Corn	36.00	25.00	12.00	0.00
Barley	0.00	16.00	33.00	48.00
Corn bran	15.00	15.00	15.00	15.00
Cottonseed meal	6.00	6.00	6.00	6.00
Soybean meal	8.00	8.00	8.00	8.00
Calcium bicarbonate	0.60	0.60	0.60	0.60
Corn gluten feed	12.00	7.00	3.00	0.00
Molasses	4.00	4.00	4.00	4.00
Limestone	1.20	1.20	1.20	1.20
Salt	0.70	0.70	0.70	0.70
Expanding urea	0.50	0.50	0.50	0.50
Premix^b^	1.00	1.00	1.00	1.00
Total	100.00	100.00	100.00	100.00
Nutrient content^c^(% DM basis)				
DM	91.48	91.40	91.32	91.81
OM	93.33	93.15	93.05	93.14
CP	17.04	17.08	16.94	17.13
NDF	44.43	41.54	40.31	41.61
ADF	13.42	13.04	14.20	14.00
Starch	25.58	25.59	25.50	25.54
GE (MJ/kg)	17.62	17.42	17.47	17.23

a *Dietary Barley Levels Defined by Its Proportion of Starch in Diets: BS-0, 0% Starch in Diets Were Provided by Barley; BS-33, 33% Starch in Diets Were Provided by Barley; BS-67, 67% Starch in Diets Were Provided by Barley; BS-100, 100% Starch in Diets Were Provided by Barley*.

b *The Premix Provided the Following per kg of Diets:Fe 25 mg,Mn 40 mg,Zn 40 mg,Cu 8 mg,I 0.3 mg, Se 0.2 mg, Co 0.1 mg, V_A_ 940 IU, V_D_ 111 IU, VE 20 IU*.

c *DM, dry matter; OM, organic matter; CP, crude protein; NDF, neutral detergent fiber; ADF, acid detergent fiber; GE, gross energy*.

### Data and Sample Collection

The amount of feed offered (4% of body weight, as feed basis) and refused were recorded to calculate the dry matter intake (DMI) every 21 days. All lambs were weighed before morning feeding on day 1 and day 63 of the fattening period to calculate ADG, and feed to gain ratio (F/G). Feed and orts samples were collected every 21 days and mixed to determine the chemical composition. During days 51–57, 6 *Hu* sheep from each treatment were selected randomly to determine apparent nutrient digestibility, with 2 days for adaptation and 4 days for sample collection. Feces samples were collected from the rectum of sheep at 0700 and 1700 h every day and were dried at 65°C and grounded through 1 mm screen. Approximately 25 g of fresh fecal were placed in a brown bottle and added 10% HCl immediately to save the nitrogen.

On day 64, 10 sheep from each treatment were euthanized to collecting rumen contents after 3 h feeding in the morning. Rumen fluids were collected and filtered through 4 layers of cheesecloth and, with the filtrates divided into three portions in 10-ml sterile tubes and stored at −20°C for the determination of volatile fatty acids (VFA), NH_3_-N, and lactic acid. After filtration, 4 ml of rumen fluid was mixed with 1 ml 25% metaphosphoric acid to determine the VFA. Rumen contents were mixed thoroughly and stored at −80°C for the bacterial DNA extraction.

### Analysis of Diet and Fecal Samples

The DM, OM, ether extract (EE, method 2003.05, AOAC International, 2000), and CP (Kjeldahl method 988.05; AOAC International, 2000) of feed, orts, and fecal samples were analyzed according to the procedures described by AOAC (14). The NDF and acid detergent fiber (ADF) were determined with α-amylase treatment and the addition of sodium sulfite (15). The starch of feed, orts, and fecal samples was analyzed using a commercial assay kit (Jiancheng Bioengineering Institute, Nanjing, China). The gross energy (GE) of feed and fecal samples was analyzed using a calorimeter (IKA-C3000, Isoperibol, Staufen, Germany). The method of acid-insoluble ash (16) was used for the nutrient digestibility measurement and calculation.

### Measurement of Ruminal Fermentation Parameters

Collected rumen fluid samples were thawed on ice, centrifuged at 2,500 × g at 4°C for 5 min, and 5 ml supernatants were subsampled. Thereafter, the subsampled ruminal fluids were used for VFA determination by the gas chromatography (TRACE 1300, Thermo Scientific, Milan, Italy) with a 30 m × 0.32 mm × 0.25 μm silica capillary column (FFAP, Agilent Technologies Co, Santa Clara, CA, USA) as described by Zhang et al. (17), using crotonic acid (19.36 mg/g) as internal standard. The concentration of lactic acid in the ruminal fluid was analyzed following the instructions of the lactic acid determination kit (A019-2-1, Jiancheng Bioengineering Institute, Nanjing, China). The NH_3_-N in the rumen fluid was analyzed following the method of Zhang et al. (18).

### Bacterial DNA Extraction and PCR Amplification

Rumen content samples were thawed, and 200 mg of each sample were used to extract the total microbial genomic DNA using an E.Z.N.A. Stool DNA kit (Omega Bio-Tek, Inc Norcross, GA, USA), following the instruction of manufacturer. The obtained bacterial DNA samples were used as templates for the quantitative real-time PCR. Primers used in the current study were based on published literatures (Table 2). Relative quantification analysis of the microorganisms in the rumen content was conducted following the method described by Li et al. (19) with using a Bio-Rad CFX96 Real-Time System (Hercules, Bio-Rad Laboratories, CA, USA).

**Table 2:** Primers used for quantitative real time-PCR amplification primer of ruminal bacteria.

**Species**	**Primer sequences((5′-3′)**	**References**
*Prevotella brevis*	F^a^:GGTTCTGAGAGGAAGGTCCCC	(13)
	R^b^:TCCTGCACGCTACTTGGCTG	
*Selenomonas ruminantium*	F:CAATAAGCATTCCGCCTGGG	(43)
	R:TTCACTCAATGTCAAGCCCTGG	
*Ruminococcus albus*	F:CCCTAAAAGCAGTCTTAGTTGG	(39)
	R:CCTCCTTGCGGTTAGAAC	
*Ruminococcus flavefaciens*	F:CGAACGGAGATAATTTGAGTTTACTTAGG	(13)
	R:CGGTCTCTGTATGTTATGAGGTATTACC	
*Fibrobacter succinogenes*	F: GGTATGGGATGAGCTTGC	(42)
		
	R: GCCTGCCCCTGAACTATC	
*Butyrivibrio fibrisolvens*	F:TAACATGAGAGTTTGATCCTGGCTC	(41)
	R:CGTTACTCACCCGTCCCGC	
Total Bacteria	F:TCCTACGGGAGGCAGCAGT	(40)
	R:GGACTACCAGGGTATCTAATCCTGTT	

a *F represents forward primers*,

b *R represents reverse primers*.

### Measurement of Dynamic Ruminal pH

Sixteen ruminal cannulated *Hu* sheep were divided into four groups (BS-0, BS-33, BS-67, and BS-100) to measure the rumen dynamic changes of ruminal pH. The experiment included 27 days (7 days for diet transition, 14 days for diet adaptation, and 6 days for data collection). The ruminal pH of each sheep was continuously monitored for 96 h from day 22 (0700 h) to day 26 (0700 h), using an industrial electrode (IP-600-9PT, Jenco, CA, USA) with a pH transmitter (692, Jenco, CA, USA). Data were recorded by a paperless recorder (SIN-R5000C, Hangzhou, China), as described in detail by Li et al. (6). The ruminal pH data for each sheep were collected every 30 s and averaged every 1 min. Ruminal pH data were calculated for every 24 h period as the mean pH, area, and time spent under the pH of 5.8 or 5.6, respectively.

### Statistical Analysis

Statistical analyses were performed using the SPSS 25.0 software (SPSS, Chicago, IL, USA) with diet as the main effect. The results of performance, nutrient digestibility, rumen fermentation parameters, rumen bacterial number, and ruminal pH data were presented as the means with their standard errors. The linear and quadratic effects of dietary BS among treatments were analyzed using polynomial regression. The comparison of different groups was performed by one-way ANOVA, followed by Duncan's *post-hoc* test for multiple comparisons. A significant difference between treatments was declared when *p* ≤ 0.05, and the trend was considered when 0.05 < *p* < 0.10.

## Results

The final BW of sheep had a tendency leading to a linear decrease (*p* = 0.09) with increasing dietary BS; while the BW gain (BWG) linearly decreased (*p* < 0.05), with lower BWG (*p* < 0.05) during the experimental period in BS-100 than that in BS-0 and BS-33 (Table 3) The DMI did not differ among treatments; however, the ADG linearly decreased (*p* < 0.05) with increasing dietary BS contents leading to a linearly increase (*p* < 0.05) for F/G. Therefore, lower (*p* < 0.05) ADG but greater (*p* < 0.05) F/G was observed in BS-100 than that in BS-0, which had similar BWG, ADG, and F/G with BS-33 and BS-67.

**Table 3:** Effects of dietary barley starch proportion on performance of fattening Hu sheep (n = 18/group).

**Items**	**Treatments^1^**				**SEM**	**P** **value**	
	**BS-0**	**BS-33**	**BS-67**	**BS-100**		**L**	**Q**
Initial BW, kg	29.88	29.41	30.05	29.59	0.210	0.859	0.821
Final BW, kg	47.36	46.68	47.03	45.58	0.308	0.090	0.651
BW gain, kg	17.48^a^	17.27^a^	16.98^ab^	15.98^b^	0.271	0.044	0.691
DMI, kg/d	1.62	1.65	1.65	1.60	0.017	0.627	0.276
ADG, g/d	277.48^a^	274.11^ab^	269.58^ab^	253.77^b^	4.314	0.044	0.690
F/G	5.89^b^	6.11^ab^	6.17^ab^	6.36^a^	0.070	0.025	0.650

1 *Dietary Barley Levels Defined by Its Proportion of Starch in Diets: BS-0, 0% Starch in Diets Were Provided by Barley; BS-33, 33% Starch in Diets Were Provided by Barley; BS-67, 67% Starch in Diets Were Provided by Barley; BS-100, 100% Starch in Diets Were Provided by Barley*.

a,b *Mean in the same row with different superscripts are different (p < 0.05)*.

*SEM, standard error of means; T, treatment; L, linear; Q, quadratic; BW, body weight; DMI, dry matter intake; ADG, average daily gain; F/G, feed to gain ratio*.

The apparent total tract digestibility of DM, OM, NDF, ADF, starch, and GE of feed linearly declined (*p* < 0.05) with increasing dietary BS proportions, whereas the digestibility of CP was not affected (*p* > 0.10) (Table 4). There were no differences (*p* > 0.10) in the digestibility of DM, OM, and starch among BS-0, BS-33, and BS-67, which were greater than that in BS-100. The digestibility of CP was not affected (*p* > 0.05) by dietary BS contents. The digestibility of NDF, ADF, and GE and the digestive energy of feed were the greatest (*p* < 0.05) in BS-0, intermediate in BS-33 and BS-67, and the lowest in BS-100.

**Table 4:** Effects of dietary barley starch proportions on nutrient apparent digestibility of fattening *Hu* sheep (*n* = 6/group, %).

**Items**	**Treatments^1^**				**SEM**	***P*** **value**	
	**BS-0**	**BS-33**	**BS-67**	**BS-100**		**L**	**Q**
DM	72.55^a^	69.89^a^	70.70^a^	64.87^b^	0.744	<0.001	0.293
OM	74.30^a^	72.70^a^	72.79^a^	67.39^b^	0.666	<0.001	0.128
CP	78.92	78.41	79.46	77.31	0.464	0.457	0.383
NDF	66.26^a^	60.34^b^	60.81^b^	53.58^c^	1.109	<0.001	0.808
ADF	46.25^a^	39.71^b^	41.69^b^	28.80^c^	1.618	<0.001	0.322
Starch	92.28^a^	92.27^a^	92.84^a^	90.50^b^	0.311	0.026	0.062
GE	74.52^a^	70.96^b^	71.82^b^	65.59^c^	0.853	<0.001	0.533
Digestive energy (MJ/kg)	13.13^a^	12.36^b^	12.54^b^	11.30^c^	0.135	<0.001	0.574

1 *Dietary Barley Levels Defined by Its Proportion of Starch in Diets: BS-0, 0% Starch in Diets Were Provided by Barley; BS-33, 33% Starch in Diets Were Provided by Barley; BS-67, 67% Starch in Diets Were Provided by Barley; BS-100, 100% Starch in Diets Were Provided by Barley*.

a,b,c *Mean in the same row with different superscripts are different (p < 0.05)*.

*SEM, standard error of means; T, treatment; L, linear; Q, quadratic; DM, dry matter; OM, organic matter; CP, crude protein; NDF, neutral detergent fiber; ADF, acid detergent fiber; GE, gross energy*.

The concentration of VFA, NH_3_-N, and lactic acid, as well as the molar ratios of isobutyrate, isovalerate, and valerate were not affected (*p* > 0.10) by the dietary BS proportions (Table 5). The molar proportion of acetate linearly decreased (*p* < 0.05) while the molar proportion of propionate linearly increased (*p* < 0.05) with increasing dietary BS contents, so that a linearly decreased (*p* < 0.05) ratio of acetate to propionate (A:P). The molar proportion of acetate and A:P in BS-0 and BS-33 was greater (*p* < 0.05) than those in BS-67 and BS-100, whereas, opposite results were observed for propionate. The molar proportion of butyrate tended (0.05 < *p* < 0.10) to be lower in BS-67 and BS-100 than in BS-33.

**Table 5:** Effects of dietary barley starch proportions on rumen fermentation parameters of fattening *Hu* sheep (*n* = 10/group).

**Items**	**Treatments^1^**				**SEM**	***P*** **value**	
	**BS-0**	**BS-33**	**BS-67**	**BS-100**		**L**	**Q**
TVFA, mM	99.75	106.55	99.55	94.75	5.354	0.832	0.614
VFA, mol/100 mol							
Acetate (A)	63.43^a^	63.74^a^	55.59^b^	50.71^b^	1.792	0.004	0.391
Propionate (P)	22.43^b^	19.57^b^	32.97^a^	35.63^a^	2.010	0.006	0.384
Isobutyrate	0.658	0.463	0.532	1.287	0.168	0.400	0.163
Butyrate	11.32	14.66	8.72	10.01	0.753	0.065	0.440
Isovalerate	1.16	0.75	0.83	0.97	0.085	0.301	0.014
Valerate	1.01	0.97	1.34	1.39	0.090	0.214	0.835
A:P	3.09^a^	3.41^a^	1.85^b^	1.78^b^	0.233	0.005	0.620
NH_3_-N, mg/ dL	2.90	3.01	3.06	2.92	0.916	0.923	0.549
Lactic acid, mg/dL	0.37	0.40	0.37	0.34	0.048	0.972	0.725

1 *Dietary Barley Levels Defined by Its Proportion of Starch in Diets: BS-0, 0% Starch in Diets Were Provided by Barley; BS-33, 33% Starch in Diets Were Provided by Barley; BS-67, 67% Starch in Diets Were Provided by Barley; BS-100, 100% Starch in Diets Were Provided by Barley*.

a,b *Mean in the same row with different superscripts are different (p < 0.05)*.

*SEM, standard error of the sample means; T, treatment; L, linear; Q, quadratic; TVFA, total volatile fatty acids*.

The quantitative PCR analysis showed that the BS levels did not affect (*p* < 0.10) the amount of ruminal *Prevotella brevis, Ruminococcus flavefaciens, Butyrivibrio fibrisolvens*, and total bacteria (Table 6). The relative abundance of *Selenomonas ruminantium* and *Ruminococcus albus* increased (*p* < 0.05) and decreased (*p* < 0.05) linearly, respectively, with increasing dietary BS proportions. The relative abundance of *Selenomonas ruminantium* in BS-67 was greater (*p* < 0.05) than that in BS-0 and BS-67; while the amount of *Ruminococcus albus* in BS-100 was lower (*p* < 0.05) than that in BS-0 and BS-33. The relative abundance of *Fibrobacter succinogenes* quadratically (*p* < 0.05) changed with diet BS proportion, with the relative abundance of *Fibrobacter succinogenes* tends (0.05 < *p* < 0.10) to be lower in BS-67 than in BS-0 and BS-100.

**Table 6:** Effects of dietary barley starch proportions on the relative abundances of rumen bacteria (*n* = 10).

**Bacteria, log10,16S rRNA copy number/g rumen content**	**Treatment^1^**				**SEM**	***P*** **value**	
	**BS-0**	**BS-33**	**BS-67**	**BS-100**		**L**	**Q**
*Prevotella brevis*	11.46	11.33	11.57	11.31	0.069	0.749	0.622
*Selenomonas ruminantium*	9.89^b^	9.97^b^	10.66^a^	10.20^ab^	0.115	0.049	0.112
*Ruminococcus albus*	10.64^a^	10.75^a^	9.69^ab^	9.20^b^	0.353	0.003	0.394
*Ruminococcus flavefaciens*	10.07	10.21	10.26	9.87	0.148	0.696	0.363
*Fibrobacter succinogenes*	9.39	8.29	8.00	9.47	0.173	0.992	0.009
*Butyrivibrio fibrisolvens*	9.34	8.85	9.16	8.58	0.132	0.145	0.855
Total bacteria	15.37	15.34	15.53	15.27	0.079	0.869	0.445

1 *Dietary Barley Levels Defined by Its Proportion of Starch in Diets: BS-0, 0% Starch in Diets Were Provided by Barley; BS-33, 33% Starch in Diets Were Provided by Barley; BS-67, 67% Starch in Diets Were Provided by Barley; BS-100, 100% Starch in Diets Were Provided by Barley*.

a,b *Mean in the same row with different superscripts are different (p < 0.05)*.

*SEM, standard error of means; T, treatment; L, linear; Q, quadratic*.

The ruminal mean pH linearly decreased (*p* < 0.05) with increasing the diet BS content proportions, with lower value in BS-100 than that in BS-0 and BS-33; however, the maximum (Max) and minimum (Min) pH value were not affected by the dietary BS proportion (Table 7). The time and area of ruminal pH below 5.8 or 5.6 increased (*p* < 0.05) linearly as the dietary BS proportion increased. The time and area of ruminal pH below 5.8 in the BS-100 and BS-67 were higher than that in the BS-0 group and had no difference with the BS-33 group (*p* < 0.05). The time and area of ruminal pH below 5.6 in the BS-100 group were greater (*p* < 0.05) than that in the BS-0, BS-33, and BS-67 groups (*p* < 0.05).

**Table 7:** Effects of dietary barley starch proportions ruminal pH parameters.

**pH**	**Treatment^1^**				**SEM**	**P-value**	
	**BS-0**	**BS-33**	**BS-67**	**BS-100**		**L**	**Q**
Mean	6.26^a^	6.06^a^	5.85^ab^	5.50^b^	0.093	0.010	0.642
Max	7.02	7.55	6.88	6.92	0.113	0.701	0.268
Min	5.73	4.57	4.57	4.73	0.246	0.176	0.183
<5.8, h/d	3.71^b^	9.80^ab^	11.95^a^	16.56^a^	1.414	0.002	0.760
<5.6, h/d	0.92^b^	5.85^b^	7.20^b^	13.92^a^	1.302	<0.001	0.674
Area under 5.8, pH × h/d	21.51^b^	56.86^ab^	69.30^a^	96.04^a^	81.99	0.002	0.760
Area under 5.6, pH × h/d	5.18^b^	32.77^b^	40.30^b^	77.93^a^	7.29	<0.001	0.674

1 *Dietary Barley Levels Defined by Its Proportion of Starch in Diets: BS-0, 0% Starch in Diets Were Provided by Barley; BS-33, 33% Starch in Diets Were Provided by Barley; BS-67, 67% Starch in Diets Were Provided by Barley; BS-100, 100% Starch in Diets Were Provided by Barley*.

a,b *Mean in the same row with different superscripts are different (p < 0.05)*.

*SEM, standard error of means; T, treatment; L, linear; Q, quadratic*.

## Discussion

### Growth Performance and Total Tract Nutrients Digestibility

In the present study, the experimental diets were formulated with identical starch content and had similar GE, and were pelleted under the same condition, which explained that the DMI of fattening *Hu* sheep was not affected by dietary BS contents. Similarly, Li et al. (6) also found that the DMI of the dairy goats was not affected by the dietary wheat proportions when the contents of starch and CP were similar among treatments. In addition, all lambs had similar initial BW, which intend to have similar rumen volume (20, 21). Therefore, it also resulted in similar DMI among treatments. The BWG and ADG were decreased, while F/G was increased in BS-100 group compared with BS-0 group with increasing dietary BS, which was inconsistent with previous studies. Petit (22) reported that changed diet grain from corn to barley decreased ADG from 362 to 332 g/day for Suffolk and Hampshire crossbred lambs. Haddad and Nasr (11) also found that increased dietary barley from 61 to 81.4% decreased the ADG from 186 to 148 g/day, and increased F/G from 4.6 to 5.2 for fattening lambs. In this study, the declined ADG and increased F/G were mainly attributed to the decreased total tract nutrients and GE digestibility when the lambs received high barley diet. These results indicated that total replacing corn grain with barley grain had negative effects on the growth performance (ADG and F/G) of fattening *Hu* lambs.

The total tract digestibility of DM, OM, and GE decreased linearly as the BS increased, which was mainly due to decreased digestibility of NDF, ADF, and starch in the present study. Haddad and Nasr (11) reported that the NDF digestibility was decreased with increasing dietary barley proportions. Leddin et al. (23) also found that increasing the amount of dietary wheat from 0 to 36% reduced the digestibility of NDF and ADF. The reduction of NDF and ADF digestibility in the total tract was usually associated with the decreased ruminal pH (24, 25) because the dramatically reduced rumen pH would inhibit the activity of cellulolytic bacteria in the rumen. In the present study, the ruminal mean pH decreased with increasing dietary BS proportions, and the time of ruminal pH under 5.6 in BS-33 BS-67 and BS-100 treatments exceed the threshold (3 h/day) of SARA suggested by Zhang et al. (26) and Castillo-Lopez et al. (27). The low ruminal pH decreased the number of cellulolytic bacteria *Ruminococcus albus*, and furtherly resulted in the declined digestibility of NDF and ADF in these treatments. The total tract starch digestibility was not affected when substituting corn with barley up to 67%; however, starch digestibility decreased when total CS in the diet was substituted with BS (92.28 vs. 90.50%). The reason for decreased starch digestibility in BS-100 could be the sharply reduced ruminal pH, resulting in subacute rumen acidosis in the lambs, which thereby limited starch degradation in rumen and intestine (28, 29). However, several studies indicated that increasing dietary barely grain improved the total tract starch digestibility because of its high rumen degradation rate (5, 30). The inconsistent results among studies were likely due to differences in diet treatments, dietary starch contents, and experimental animals. For example, Johnson et al. (5) reported a greater starch digestibility for barley-based diet than that for corn-based diet at a dietary starch content (ranged from 55.4 to 62.25%) was reported in beef cattle. The current results indicated that partially substitute CS with BS could decrease total tract fiber digestibility for fattening sheep, and total substitute CS with BS would decrease both fiber and starch digestibility for fattening sheep.

### Rumen Fermentation

Although the digestibility of NDF and ADF was decreased in diets containing BS, the concentration of total TVFA (TVFA) was similar among treatments. However, the fermentation pattern was different greatly for diets mainly containing CS and BS, with fermentation switched from acetate to propionate as the dietary BS contents increased. These results agreed with Li et al. (6), who observed that replacing dietary corn with wheat (0, 17.5, and 35.0% of DM) did not affect the concentration of ruminal TVFA because of similar starch contents among treatments. Meanwhile, Johnson et al. (5) also reported that the proportion of ruminal propionate was increased from 42.17 to 46.51 mol/100 mol as the ratio of barely increased. The increased molar proportion of propionate was consistent with the enhanced relative abundance of *Selenomonas ruminantium*, which is reported as starch degradation bacteria that produces propionate (31) in BS-67 and BS-100 compared with that in BS-0. On the contrast, the decreased proportion of acetate in BS-67 and BS-100 groups was attributed to the reduced NDF digestibility (32). This was accomplished by ruminal cellulolytic bacteria that decompose the fiber part of the diet (33). In our study, the lower relative abundance of *Ruminococcus albus* in BS-100 than that in the BS-0 and BS-33, which was consistent with the result of acetate.

### Rumen Bacterial Community

It is well accepted that ruminants intake high starch diet could reduce the abundances of cellulolytic bacteria due to the decrease of ruminal pH (34, 35). In the present study, the ruminal pH decreased with increasing dietary BS proportion, and the duration of ruminal pH below 5.6 exceeds 3 h/day in BS-67 and BS-100. The lower ruminal pH in the BS-67 and BS-100 indicated that sheep were experiencing SARA (36), which led to a decreasing amount of ruminal *Ruminococcus albus*. However, the dietary treatments did not affect the amount of ruminal *Ruminococcus flavefaciens, Fibrobacter succinogenes*, and *Butyrivibrio fibrisolvens*. These results are different from those results of Khafipour et al. (36) and Li et al. (6), who reported that these bacterial populations declined when the dairy cow and dairy goat received a high grain diet. The mainly cellulolytic bacteria, such as *Ruminococcus flavefaciens, Fibrobacter succinogenes*, and *Butyrivibrio fibrisolvens*, were believed to be sensitive to pH (37); however, the amount of these bacteria was not declined with increasing dietary BS.

In the present study, the amount of *Selenomonas ruminantium* was improved with the increasing of dietary BS proportions, while the amount of *Prevotella brevis* was unchanged. Tajima et al. (38) found the quantity of *Selenomonas ruminantium* was dramatically increased 3 days after the diet changed and was double numbered on the day 28, while the amount of *Prevotella* species was increased in 3 days and approached initial values in 28 days when the cattle switched from a diet containing 21% grain to a diet containing 82% grain. This study indicated the *Selenomonas ruminantium* could adapt to the high grain diet, but the *Prevotella brevis* showed a short-term adaptation to this type of diet.

## Conclusions

In conclusion, feeding a high-concentrate corn-based diet for fattening *Hu* sheep improved the performance and rumen fermentation parameters when compared to a barley-based diet. Increasing dietary BS levels linearly decreased ruminal mean pH and increased time of pH below 5.80 and 5.60 that resulted in a higher SARA risk. The recommended substitution of CS with BS in the diet is 67%, which can save feed cost without compromising the performance of sheep as well as NDF and starch degradability.

## Data Availability Statement

The original contributions presented in the study are included in the article/supplementary material, further inquiries can be directed to the corresponding author.

## Ethics Statement

The animal study was reviewed and approved by the Biological Studies Animal Care and Use Committee of Gansu Province, China (2005-12).

## Author Contributions

XM collected the sample, analyzed the data, and drafted the manuscript. WZ, TR, ZZ, TG, FaL, and LG collected the sample. FeL presented the idea of this manuscript, supported the funding, analyzed the conclusions, and revised the manuscript. All authors contributed to the article and approved the submitted version.

## Funding

This research was financially supported by the National Natural Science Foundation of China (32072754), and the Natural Science Foundation of Gansu Province, China (20JR10RA299).

## Conflict of Interest

The authors declare that the research was conducted in the absence of any commercial or financial relationships that could be construed as a potential conflict of interest.

## Publisher's Note

All claims expressed in this article are solely those of the authors and do not necessarily represent those of their affiliated organizations, or those of the publisher, the editors and the reviewers. Any product that may be evaluated in this article, or claim that may be made by its manufacturer, is not guaranteed or endorsed by the publisher.
